# Prognostic significance of serum osteopontin levels in small cell lung cancer

**DOI:** 10.1186/s12890-020-01242-3

**Published:** 2020-09-01

**Authors:** Chunhua Xu, Qi Yuan, Wei Wang, Chuanzhen Chi, Qian Zhang, Li Li, Rusong Yang, Yuchao Wang

**Affiliations:** 1 Department of Respiratory Medicine, the Affiliated Brain Hospital of Nanjing Medical University, 215 Guangzhou Road, Nanjing, 210029 Jiangsu China; 2 The Clinical Center of Nanjing Respiratory Diseases and Imaging, Nanjing, 210029 Jiangsu China; 3 Department of Thoracic Surgery, the Affiliated Brain Hospital of Nanjing Medical University, 215 Guangzhou Road, Nanjing, 210029 Jiangsu China

**Keywords:** Small cell lung cancer, Osteopontin, Biomarker, Treatment responses, Overall survival

## Abstract

**Background:**

Osteopontin (OPN) is closely related to tumor occurrence and metastasis. This study explored the clinical value of serum OPN levels in small cell lung cancer (SCLC) patients.

**Methods:**

The ELISA method was used to determine the OPN level of 96 SCLC patients before and after first-line chemotherapy, and compared with 60 healthy controls.

**Results:**

The serum OPN level of SCLC patients before treatment was significantly higher than that of the healthy control (*P* < 0.001). Serum OPN levels were related to disease stage, tumor size, and lymph node metastasis (*P* = 0.012, 0.034, and 0.037, respectively). Serum OPN level decreased after first-line chemotherapy (*P* = 0.019), which was related to treatment response (*P* = 0.011). The serum OPN level was an independent predictor of overall survival.

**Conclusions:**

The serum OPN level can be used as a biomarker to predict treatment response and survival of SCLC patients.

## Background

Lung cancer is one of the most common tumors in the world. According to the pathological type, it is divided into non-small cell lung cancer (NSCLC) and small cell lung cancer (SCLC), of which SCLC accounts for 13% of the total number of cases [1, 2]. Although SCLC is sensitive to radiotherapy and chemotherapy, most of them are diagnosed at an advanced stage with a poor prognosis [3]. The median survival time of untreated patients is only 2–4 months, and the 5-year overall survival rate is 3–8%. So far, platinum-based and etoposide chemotherapy is still the first-line treatment, but the tumor is prone to relapse and metastasis, and the prognosis is poor [4–6]. Therefore, it is necessary to find new biomarkers, to discover and predict the treatment response early.

Osteopontin (OPN) is a phosphorylated glycoprotein that is involved in regulating cell adhesion, migration, and invasion [7, 8]. Previous studies have shown that many tumors, such as pancreatic cancer, colon cancer, and NSCLC, have elevated OPN levels [9–13]. Studies have shown that OPN overexpression is related to tumor progression and poor prognosis [14, 15]. Although many studies have shown that OPN is related to the prognosis of various cancers, the relationship between its expression and the clinicopathological characteristics of SCLC patients is still unclear. Therefore, this study first evaluated the serum OPN levels of SCLC patients and healthy controls, and explored the relationship between OPN levels and treatment response and overall survival. We provide OPN as a biomarker for predicting treatment response and survival in SCLC patients.

## Methods

### Patients

In this study, we collected the serum samples of 96 SCLC patients admitted to the Affiliated Brain Hospital of Nanjing Medical University. All patients were diagnosed with SCLC by histological or cytological examination. Patients undergoing surgery were excluded from the study. The SCLC patients were staged according to the Veterans Administration Lung Cancer Research Group (VASG) staging system [16]. All patients have a measurable disease through computer tomography. Patients received chemotherapy with etoposide and cisplatin (EP) or etoposide and carboplatin (EC) with or without radiotherapy. Adjust the dose according to each patient’s physical condition. Patients have regular blood chemistries, abdominal ultrasound or computed tomography, brain magnetic resonance imaging, and bone imaging.

In addition, we collected serum samples from 60 healthy controls and matched them with SCLC cases.

### Evaluation of therapy responses

All patients received 2–6 cycles of EP or EC chemotherapy, with or without radiotherapy. Tumor response was measured using solid tumor criteria 2 cycles after completion of treatment, including complete response (CR), partial response (PR), stable disease (SD), and progressive disease (PD) [17]. Patients evaluated as CR, PR, and SD continued the original chemotherapy regimen, while patients with PD changed the treatment regimen.

All patients were followed up regularly, the last follow-up time is July 1, 2019. The overall survival (OS) refers to the time from diagnosis to death or last visit.

### Enzyme linked immunosorbent assay (ELISA)

After diagnosis, blood samples are taken from the patient before treatment. After 2 cycles of treatment, if the response was effective, continue to take serum samples after 4 cycles of treatment. If they were ineffective, take serum samples immediately. The samples were centrifuged at 1500×g for 10 min, and the serum was stored at − 80 °C until analysis. Serum OPN levels were determined using an anti-OPN monoclonal antibody ELISA kit (R&D Systems, Minneapolis, MN, USA), and OPN: MDD (median detection density) was 0.22 ng/ml.

### Statistical analysis

Statistical analysis uses spssv13.0 software. The Mann-Whitney U test was used to compare the groups. The survival curve was analyzed using Kaplan-Meier curve. Cox regression model was used to analyze the relationship between OS, pathological characteristics and OPN. The receiver operating characteristic curve (ROC) was used to analyze the cut-off value of serum OPN in SCLC patients and healthy controls. Take the maximum value of the sum of specificity and sensitivity as the optimal cut-off value. *P* < 0.05 was considered statistically significant.

## Results

### Patients’ characteristics

Among 96 SCLC patients, 66 were male and 30 were female, with a median age of 55 years. 58 patients had a history of smoking, 38 had no history of smoking, 28 had limited SCLC, 58 had extensive SCLC, and 70 had lymph node metastasis. Most patients received EP chemotherapy (*n* = 80) and radiotherapy (*n* = 86). After treatment, 78 cases were CR or PR, 18 cases were SD and PD (Table 1). The median follow-up time was 12 months (2–30 months), the median OS was 11 months, the limited stage SCLC was 14 months, and the extensive SCLC was 8.5 months.

**Table 1:** The characteristics of SCLC patients and healthy controls

Variables	SCLC patients	Healthy controls	***P***
Number of subject	96	60	
Median (range)	55 (36–75)	56 (38–74)	0.618
Male/Female	66/30	40/20	0.786
Smoking history			
Ever smoker	58	32	0.384
Never smoker	38	28	
Performance status			
0–1	56		
2–3	40		
Disease stage			
Limited	28		
Extended	58		
Tumor size (cm)			
≥ 3	76		
< 3	20		
Lymph node metastasis			
N0	26		
N1–3	70		
Chemotherapy			
EP	80		
EC	16		
Radiotherapy sequence			
Concurrent	43		
After-chemotherapy	40		
None	13		
Responses			
CR + PR	78		
SD + PD	18		

EP, etoposide+platinum; EC, etoposide+cisplatin; CR, complete response; PR, partial response; PD, progressive disease; SD, stable disease

### Serum OPN levels in SCLC patients and healthy controls

**Fig. 1:**
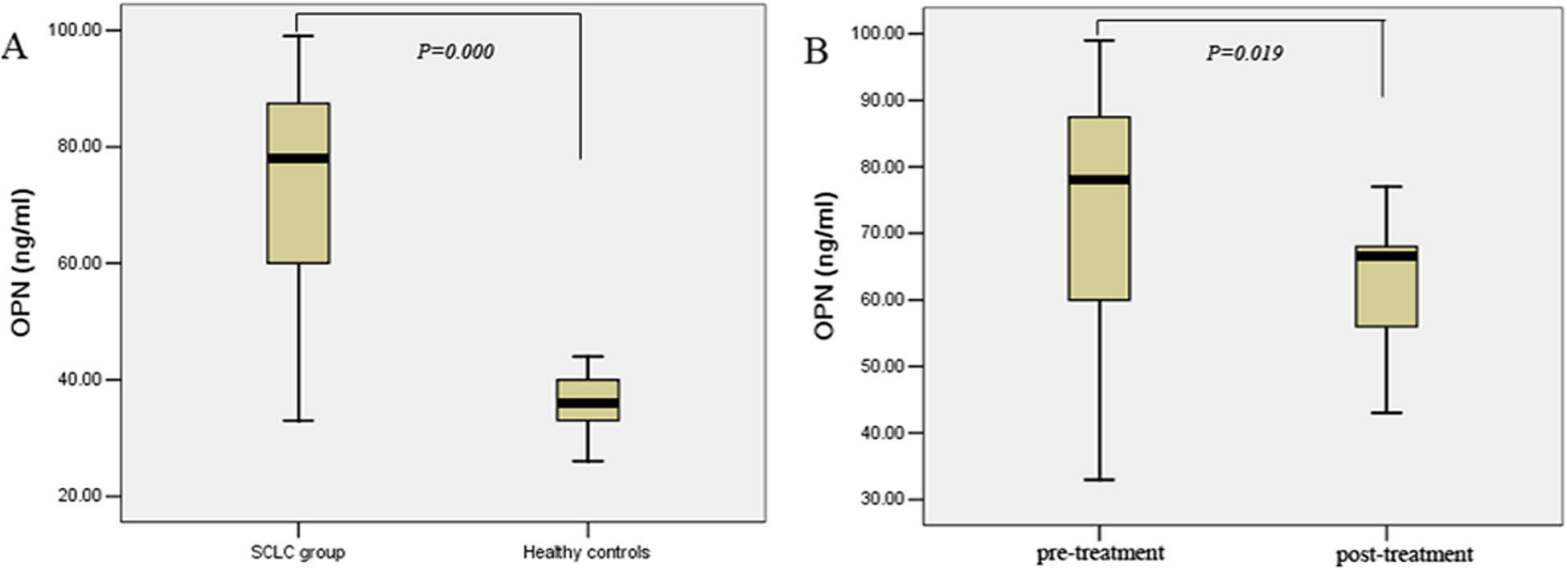
The serum levels of OPN in SCLC patients vs. healthy controls or pre-and after-treatment. (A) Patients with SCLC had higher serum OPN level than that of healthy controls (*P* = 0.000). (B) Association of pre- and after-treatment levels of serum OPN in SCLC patients (*P* = 0.019)

The serum OPN level before treatment in the SCLC group was (72.07 ± 19.09) ng/ml, while the 60 healthy controls was (36.06 ± 5.48) ng/ml, indicating that the serum OPN level of SCLC patients before treatment was significantly higher than that in the healthy controls (*P* = 0.000, Fig. 1A). Serum OPN levels decreased after chemotherapy (72.07 ± 19.09 ng/ml vs. 61.69 ± 10.42 ng/ml, *P* = 0.019, Fig. 1B).

### The relationship between pre-treatment OPN levels and clinicopathological characteristics

**Fig. 2:**
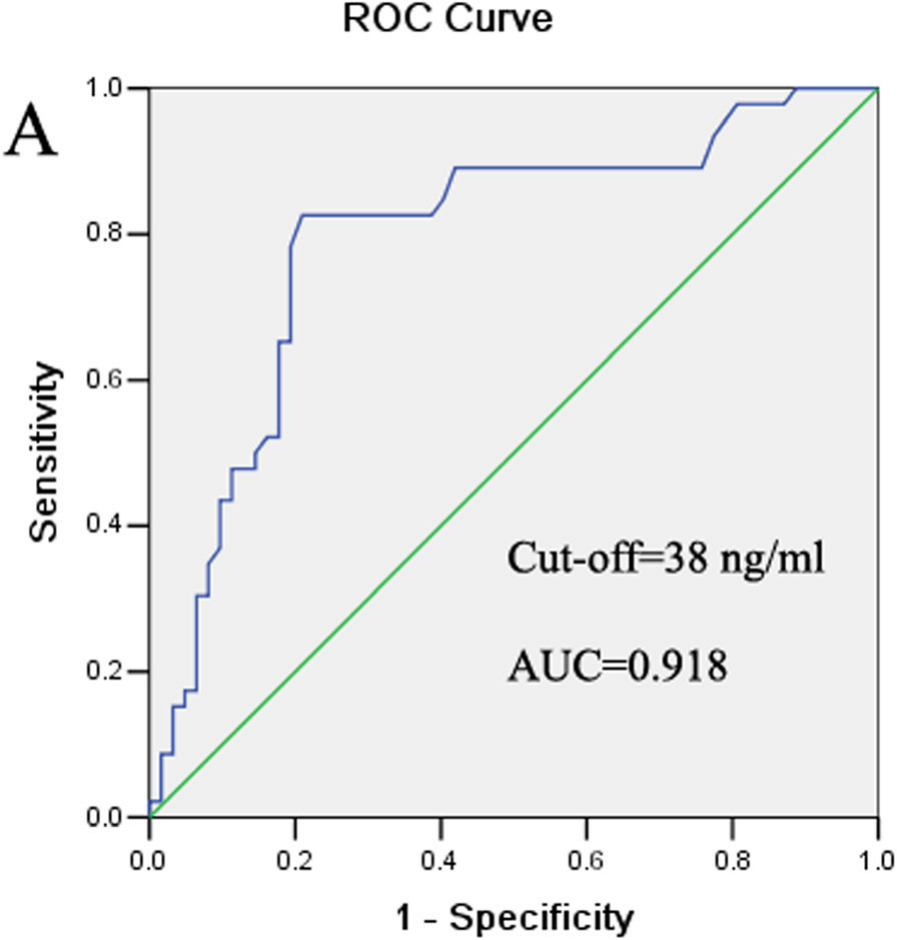
ROC curve of the serum OPN levels for differentiation between SCLC patients and healthy controls

The ROC curve was calculated based on the serum OPN levels of 96 SCLC patients before treatment and 60 healthy controls (Fig. 2). The estimated area under the ROC curve was 0.918. The optimal cut-off value for serum OPN level was 38 ng/ml. We analyzed the relationship between OPN level and the clinicopathological characteristics of SCLC patients. OPN level was related to disease stage (*P* = 0.012), tumor size (*P* = 0.034), and lymph node metastasis (*P* = 0.037), while there were no difference with age (*P* = 0.954), gender (*P* = 0.317), smoking status (*P* = 0.077), performance status (*P* = 0.174), and chemotherapy responses (*P* = 0.485, Table 2).

**Table 2:** Association between serum OPN levels and clinicopathological characteristics in SCLC patients

Characteristics	Number	OPN level (ng/ml)	***P***
Age, yr			0.954
≥ 60	54	73.78 ± 16.55	
< 60	42	73.43 ± 17.90	
Gender			0.317
Male	66	75.38 ± 16.44	
Female	30	68.38 ± 18.19	
Smoking history			0.077
Ever smoker	58	68.32 ± 20.11	
Never smoker	38	78.94 ± 11.13	
Performance status			0.174
0–1	56	70.02 ± 17.39	
2–3	40	78.27 ± 15.55	
Disease stage			0.012
Limited	28	66.32 ± 19.64	
Extended	58	80.94 ± 9.41	
Tumor size (cm)			0.034
≥ 3	76	77.96 ± 14.31	
< 3	20	60.63 ± 18.18	
Lymph node metastasis			0.037
N0	26	64.08 ± 21.85	
N1–3	70	79.40 ± 9.72	
Responses			0.485
CR + PR	78	71.15 ± 18.33	
SD + PD	18	78.01 ± 13.74	

OPN, osteopontin; CR, complete response; PR, partial response; SD, stable disease; PD, progressive disease

### Relationship between serum OPN levels and treatment response

**Fig. 3:**
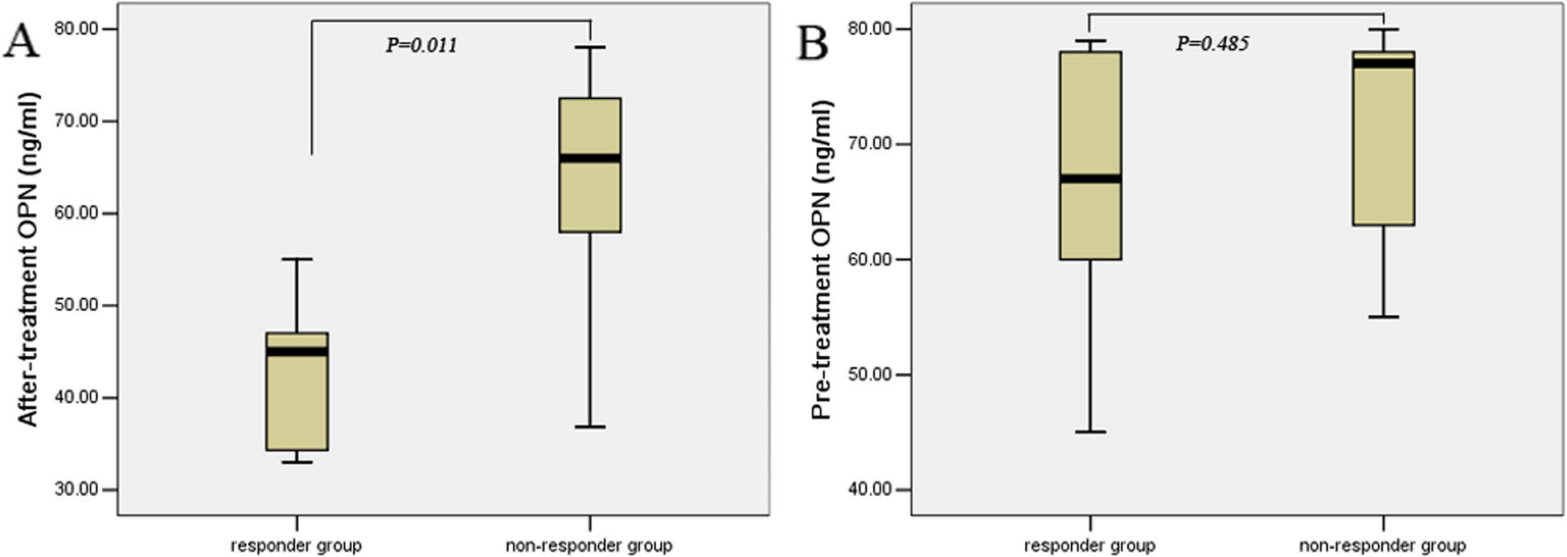
Association of pre- and after-treatment levels of serum OPN in SCLC patients. (A) Association of after-treatment levels of serum OPN in SCLC patients, with responders vs. non-responders (*P* = 0.011). (B) Association of pretreatment levels of serum OPN in SCLC patients with responders vs. non-responders (*P* = 0.485)

After radiotherapy and chemotherapy, the objective response rate (CR + PR) was 81.2%, and the non-response rate (SD + PD) was 18.8%. The serum OPN of chemotherapy-sensitive patients was significantly lower than that of non-responders (48.36 ± 12.18 ng/ml vs. 72.19 ± 11.48 ng/ml, *P* = 0.011, Fig. 3A). Our results showed that serum OPN levels were significantly correlated with treatment response. However, there was no significant correlation between serum OPN levels and treatment response before treatment (71.15 ± 18.33 ng/ml vs. 78.01 ± 13.74 ng/ml*, P* = 0.485, Fig. 3B).

### Prognostic value of serum OPN levels for SCLC patients

We used univariate and multivariate analysis to predict prognostic factors in patients with OS. Our univariate analysis data showed that performance status, disease stage, and serum OPN levels after treatment were prognostic factors for OS. Our multivariate analysis showed that performance status, disease stage, and serum OPN levels after treatment were prognostic factors for OS. However, the serum OPN levels of these patients before treatment were not related to OS (Table 3).

**Table 3:** Univariate and multivariate Cox analysis of variables considered for OS of SCLC patients

Characteristics	Univariate			Multivariate		
	HR	95% CI	***P***	HR	95% CI	***P***
Gender (Male vs. Female)	3.563	0.496–25.582	0.206	2.264	0.382–13.404	0.368
Age (< 60 vs. ≥60)	0.819	0.140–4.806	0.825	2.091	0.514–8.502	0.302
Disease stage (Limited vs. Extended)	4.277	1.233–14.835	0.022	3.429	1.096–10.726	0.034
Lymph node metastasis (N_0_ vs. N_1–3_)	0.585	0.157–2.182	0.424	2.358	0.606–9.181	0.216
PS (0–1 vs. 2–3)	4.820	1.196–19.424	0.027	4.004	1.281–12.513	0.017
Smoking history (Ever vs. Never)	1.719	0.551–5.365	0.351	0.649	0.218–1.933	0.438
Pre-OPN (Negative vs. Positive)	2.662	0.908–7.807	0.075	0.305	0.091–1.106	0.053
After-OPN (Negative vs. Positive)	4.936	1.793–13.586	0.002	6.114	1.661–22.510	0.006

CI, confidence interval; HR, hazard ratio; PS, performance status; OPN, osteopontin; OS, overall survival

**Fig. 4:**
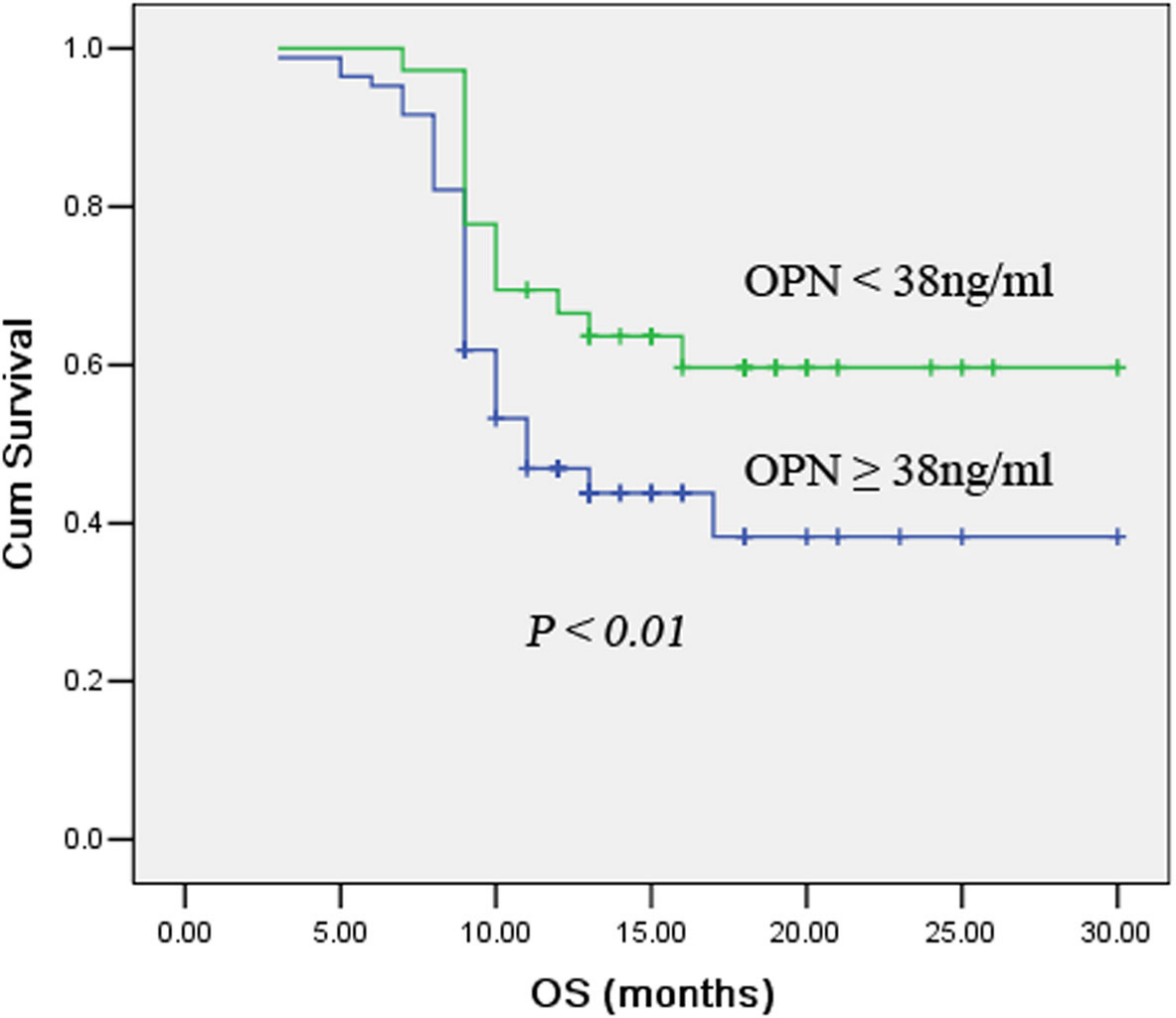
Kaplan–Meier curves stratified by the after-treatment levels of serum OPN. Log-rank test determined that the OS in low OPN group were significantly longer than those in the high OPN group (*P* < 0.05)

We also used Kaplan-Meier curve and log-rank test to analyze the relationship between serum OPN levels and OS after treatment in SCLC patients, and found that SCLC patients with reduced serum OPN levels after treatment had better OS (Fig. 4).

## Discussion

SCLC is a highly malignant lung tumor, although progress has been made in early detection and improvement of treatment methods [18]. This study found that the serum OPN level of SCLC patients was higher than that of the healthy controls, and the serum OPN level decreased after treatment. Therefore, the serum OPN level can be used as an effective biomarker to evaluate the therapeutic efficacy of SCLC patients.

OPN is a phosphorylated glycoprotein, which is closely related to the growth, migration and invasion of tumor cells [19]. Many studies have shown that the abnormal expression of OPN is closely related to the occurrence and development of liver cancer, colon cancer and gynecological malignant tumors. The mechanism may be that OPN promotes the formation of new blood vessels, the formation of cytokines, the adhesion and chemotaxis of extracellular matrix, and inhibits apoptosis to varying degrees [20].

OPN expression is an independent predictor of platinum first-line chemotherapy response and prognosis in patients with advanced NSCLC [21]. The survival rate of patients with low serum OPN levels was higher than that of patients with high OPN levels [22]. In SCLC, OPN reduces cisplatin-induced apoptosis and induced chemotherapy resistance [23]. In our study, we confirmed that the serum OPN level of SCLC patients was higher than that of the control group. Our results also show that serum OPN after treatment can predict the therapeutic effect of SCLC patients. All these studies have confirmed the effect of OPN expression on tumor progression and response. The overexpression of OPN in tumor tissues may be due to the rapid growth of tumor cells and the lack of proper blood supply. The tumor cells death by inducing apoptosis or necrosis, leading to the up-regulation of apoptosis-related proteins.

Previous studies have shown that OPN secretion is related to the proliferation of tumor cells [24]. This study found that serum OPN levels were related to VALSG stage, tumor size, and lymph node metastasis, suggesting that increased serum OPN levels may be related to tumor cells. In addition, our data show that serum OPN levels can predict OS in patients with SCLC.

## Conclusions

In conclusion, our study revealed the value of serum OPN levels in the prognosis of SCLC. However, before OPN can be used as a predictor of SCLC prognosis, a multicenter study with a larger sample size is needed to verify our data.

## Data Availability

The datasets analyzed during the current study are available from the corresponding author on reasonable request.

## Abbreviations

OPN: Osteopontin
SCLC: Small cell lung cancer
ELISA: Enzyme-linked immunosorbent assay
NSCLC: Non-small cell lung cancer
EP: Etoposide and cisplatin
EC: Etoposide and carboplatin
CR: Complete response
PR: Partial response
SD: Stable disease
PD: Progressive disease
OS: Overall survival
